# Nitrogen Addition Affects Nitrous Oxide Emissions of Rainfed Lucerne Grassland

**DOI:** 10.3390/ijerph19137789

**Published:** 2022-06-24

**Authors:** Yuan Li, Gang Wang, Narasinha J. Shurpali, Yuying Shen

**Affiliations:** 1 The State Key Laboratory of Grassland Agro-Ecosystems, Lanzhou University, Lanzhou 730020, China; yuanli@lzu.edu.cn (Y.L.); Wangg14@lzu.edu.cn (G.W.); 2 College of Pastoral Agri-Culture Science and Technology, Lanzhou University, Lanzhou 730020, China; 3 National Field Scientific Observation and Research Station of Grassland Agro-Ecosystems in Gansu Qingyang, Lanzhou University, Lanzhou 730020, China; 4 Grasslands and Sustainable Farming, Production Systems Unit, Natural Resources Institute Finland, Halolantie 31A, FI-71750 Kuopio, Finland; narasinha.shurpali@luke.fi

**Keywords:** precipitation variation, soil gas diffusivity, water-filled pore spaces, fertiliser nitrogen, Loess Plateau

## Abstract

Nitrous oxide (N_2_O) is a potent greenhouse gas. Assessing the N_2_O emission from lucerne grasslands with nitrogen addition will aid in estimating the annual N_2_O emissions of such agriculture areas, particularly following summer rainfall events in light of precipitation variation associated with global change. Here, we measured soil N_2_O emissions, soil temperature and water content of lucerne grasslands with four levels of nitrogen addition over 25 days, which included 10 rainfall events. Results showed that nitrogen addition was observed to increase soil NO_3_^−^-N content, but not significantly improve dry matter yield, height or leaf area index. Nitrogen addition and rainfall significantly affected N_2_O emissions, while the response of N_2_O emissions to increasing nitrogen input was not linear. Relative soil gas diffusivity (D*_p_*/D*_o_*) and water-filled pore space (WFPS) were good indicators of N_2_O diurnal dynamics, and D*_p_*/D*_o_* was able to explain slightly more of the variation in N_2_O emissions than WFPS. Collectively, nitrogen addition did not affect lucerne dry matter yield in a short term, while it induced soil N_2_O emissions when rainfall events alter soil water content, and D*_p_*/D*_o_* could be a better proxy for predicting N_2_O emissions in rainfed lucerne grasslands.

## 1. Introduction

Nitrous oxide (N_2_O) is a potent greenhouse gas in the atmosphere that participates in the destruction of the ozone in the stratosphere and has a global warming potential 265–298 times greater than that of carbon dioxide at a 100-year timescale [1]. Mitigating N_2_O emissions generated by the agricultural sector is a major challenge, as it accounts for 50% of global N_2_O emissions [1,2]. Agricultural N_2_O emissions are induced by increasing soil nitrogen availability driven by the application of nitrogen fertiliser and biological nitrogen fixation (BNF), which both have emission factors of 1.25% [1].

Most intensively managed pastures with high productivity receive applications of nitrogen fertiliser [2,3,4], including cultivated lucerne (*Medicago sativa*) grasslands, which is a major perennial legume forage in farming systems of the western Loess Plateau [5,6]. Although lucerne has active BNF capabilities, reasonable nitrogen fertiliser application can extend the growing year under sustainable utilization as the aboveground biomass is regularly harvested for forage [5,6]. However, nitrogen fertiliser application (e.g., NO_3_^−^ or ammonium) accounts for about 50% of the global anthropogenic N_2_O emissions resulting from nitrification and denitrification of soil available nitrogen [7,8,9,10,11].

N_2_O emissions in agricultural soils are produced from nitrification by ammonia-oxidizing bacteria (AOB) and archaea (AOA) under aerobic conditions, which convert ammonia, via nitrite (NO_2_^−^), to nitrate (NO_3_^−^); denitrification under anaerobic conditions sequentially reduces NO_3_^−^ to dinitrogen (N_2_), and N_2_O is a by-product [1,2,3,4,5]. Soil water content is critical for soil nitrification and denitrification, and this is particularly the case in arid and semi-arid regions where uncertain rainfall after dry periods causes soil rewetting [12,13,14,15]. Hence, N_2_O emissions in semi-arid regions such as the Loess Plateau are often greater following summer rainfall events, especially with nitrogen fertiliser application [12,13,14,15]. Water-filled pore space (WFPS) as a key proxy for soil water content is used to relate N_2_O emissions with changes in soil water content [13,16,17,18]. However, WFPS does not represent the fraction of the entire soil volume that is gas permeable and cannot express the physical force with which water is held in soil or soil pore connectivity and tortuosity [16,19], which are crucial to determining soil gas transport. Gas diffusion in the soil is related to both soil porosity and soil water content [20,21]. Relative soil gas diffusivity (D*_p_*/D*_o_*), defined as the ratio of the soil gas diffusion coefficient to the free air gas diffusion coefficient, can be an excellent factor to describe soil gas transport between the soil and the atmosphere [20,22]. D*_p_*/D*_o_* be a key predictor of N_2_O emissions even under varied soil matrix potential and bulk density levels [22,23,24,25,26,27,28].

Evaluating the effect of soil water content on N_2_O emissions in lucerne grasslands under nitrogen addition during periods of high rainfall variability will aid us in estimating annual N_2_O emissions and increase our knowledge about N_2_O emissions in dryland forage production systems. Thus, the aims of the study were as follows: (1) to measure soil N_2_O emissions from lucerne grasslands with four levels of nitrogen addition over 25 days, which included 10 periods of rainfall; (2) to relate N_2_O emissions to changes in soil water content by WFPS and D*_p_*/D*_o_*. We hypothesized that besides WFPS, D*_p_*/D*_o_* is also a strong indicator of N_2_O emissions in rainfed lucerne grassland when rainfall events alter soil water content.

## 2. Materials and Methods

### 2.1. Site Description

This study was undertaken at the Loess Plateau Research Station of Lanzhou University (35°39′ N, 107°51′ E, 1297 m above sea level), Lanzhou, China. The area is characterized by a cool continental climate, with mean annual precipitation of 548 mm and rainfall that is most abundant from July through September. The average annual temperature ranges from 8 °C to 10 °C. The mean temperatures in the warmest (July) and coldest (January) months are 21.3 °C and −5.3 °C, respectively. Soil temperature (0–100 mm) ranged from −1.6 °C (March) to 30.6 °C (August), and soil water content (0–100 mm) ranged from 7.8% (January) to 14.8% (August). The soil is classified as silty loam soil according to the FAO/UNESCO soil classification, and the soil characteristics, based on samples collected at the start of the measurement periods, are described in Table 1.

### 2.2. Experimental Design and N_2_O Flux Measurements

The experiment was conducted according to a randomized block design; each plot was 3 m wide × 3 m in length with three replicates. A 0.5-m-wide buffer zone was included between each plot. One of four nitrogen addition levels was applied to each plot, 0, 50, 100 and 150 kg N ha^−1^, and are referred to herein as N0, N50, N100 and N150. The fertiliser used was based on urea with 40% nitrogen content. Fertiliser applications occurred twice, on April 5 (recovery stage) and June 5 (after the first cutting) with 80% and 20% of the allotted fertiliser, respectively. This study was conducted from the 1st to 25th of August 2014 using lucerne crops, corresponding to when rainfall would be abundant, and the days of the experiment are thus referred to as Day 1 to Day 25.

Lucerne grasslands (*Medicago sativa* L. cv ‘Longdong’) were planted by drilling in 2009, with 300 mm row spacing and a sowing rate of 15 kg ha^−1^. The previous crop on the lucerne grasslands was maize (*Zea mays*). The Lucerne grasslands were rainfed, and 302 kg ha^−1^ of KH_2_PO_4_ was applied each year during the regreening stage. Lucerne was harvested twice at its flowering stage each year (June and July) as is typically done by growers in the region [8]. No tillage practices were performed during growing, and the average yield was 11,982 kg hm^−2^.

Measurement of N_2_O emissions occurred continually during the 25 days. Soil N_2_O concentrations were measured using an N_2_O/CO near-infrared gas analyser (Model DLT-100, Los Gatos Research, Inc., San Jose, CA, USA) connected to three replicates in closed dynamic soil respiration chambers (diameter 350 mm, height 600 mm, model SC-03, LI-CA, China) placed in each plot. The slope of the change in N_2_O concentration of the chamber headspace over the measuring time was calculated to obtain the N_2_O flux (*F;* mg m^−2^ h^−1^) using Equation (1):

(1) $$F=\frac{m_{2}-\;m_{1}}{A\times \left(t_{2}-t_{1}\right)}=\frac{C_{2}\times V\times M_{0}\times \frac{273}{T_{2}}-C_{1}\times V\times M_{0}\times \frac{273}{T_{1}}}{A\times \left(t_{2}-t_{1}\right)\times 22.4}$$

Here, *A* is the surface area (m^2^) of the chamber, *V* is the volume (L) of the chamber, *M*_0_ is the molecular weight of the N_2_O; *t*_1_ and *t*_2_ are the times in the beginning and ending of gas measurement, *m*_1_ and *m*_2_ are the masses of the N_2_O (mg) in the chamber at *t*_1_ and *t*_2_, respectively; *C*_1_ and *C*_2_ are the volume concentrations of N_2_O in the chamber at *t*_1_ and *t*_2_, respectively; and *T*_1_ and *T*_2_ are the air temperatures in the chamber at *t*_1_ and *t*_2_, respectively. The N_2_O flux was simultaneously computed with an in-built microcomputer in the N_2_O/CO near-infrared gas analyser.

The measurement accuracy of the N_2_O/CO near-infrared gas analyser is 1 ppb. Minimum detectable N_2_O fluxes for the N_2_O/CO analyser were determined by using the linear regression method described by Parkin et al. (2012) [29]. The minimum detectable flux was approximately −0.005 mg N_2_O m^−2^ h^−1^. Detection limits were used to calibrate flux data, and fluxes below the detection limit were assigned a value of zero, in total, 5% of N_2_O fluxes were assigned as 0.

Each chamber was fitted with a small fan mounted inside the lid to maintain well-mixed conditions. Three replicate chambers were used for each plot, randomly distributed in each plot and pressed 50 mm into the soil. Flux data were logged and calculated by a Soil Flux System (MCC-1-8, LI-CA, Beijing, China). The Soil Flux System was connected to a DLT-100 N_2_O/CO analyser for N_2_O flux recording. The daily dynamics of N_2_O emissions (over 24 h) were monitored overall, and each plot was measured 24 times during each 24-h cycle. At the same time, chamber temperature was measured with sensors installed inside of the chamber lid.

### 2.3. Soil Measurement and Analysis

During each gas sampling period, hourly soil temperature and soil volumetric water content (θ_v_) readings were measured by probes close to each chamber, installed at a 50 mm depth. Soil samples, at depths of 0–100, 100–200 and 200–300 mm, were taken at five sites near each chamber with a 50-mm-diameter gouge auger. Soil samples were placed in airtight plastic bags, transported to the laboratory and stored temporarily at 4 °C in the dark. The fresh soil was analysed for nitrate nitrogen (NO_3_^−^-N). Soil organic carbon (SOC) and total nitrogen (TN) were analysed using air-dried soil.

SOC was determined using the potassium dichromate/sulfuric acid mixture titration method, and TN was measured by the Kjeldahl method (Kjeltec^TM^ 8400, FOSS, Hilleroed, Denmark). NO_3_^−^-N was extracted with 1 M KCl and measured with a colorimetric continuous flow analyzer (FIAstar 5000, FOSS).

Bulk density (Mg m^−3^) of the soil at a 0–100 mm depth using a ring (50 mm internal diameter, 50 mm height) was computed as the ratio of the mass of dry soil (g) to the volume of the sample (m^3^) [30]. The average field capacity and the permanent wilting point are 0.27 and 0.8%, respectively. Soil WFPS was computed using bulk density and θ_v_ [16]. Water-induced linear reduction (WLR) with the Buckingham model well predicted gas diffusivity at relatively dry conditions and was used to calculate soil D*_p_*/D*_o_* [31].

### 2.4. Plant Measurement and Analysis

Samples were collected along two 0.5 m transects, which were made at each plot before (16 July) and at the end of the experiment (23 August) when stubble height was 50 mm. The dry matter yield (DM, kg ha^−1^) was measured after oven drying for 48 h at 65 °C.

Leaf area index (LAI) was determined by an AccuPAR/LAI canopy analyzer (LP-80, Decagon Devices, Inc., Pullman, WA, USA) with four replicates per treatment.

### 2.5. Statistical Analysis

Analysis of variance (ANOVA) was utilized to determine significant differences in soil NO_3_^−^-N, soil water content and temperature data among the nitrogen fertiliser addition treatments for each period. Comparisons of soil N_2_O emissions were made using the least significant difference test (LSD) at 5%. After confirming the normality of the data, interactions between nitrogen addition and rainfall to N_2_O emissions were analysed using two-way ANOVA. A general exponential model was used to determine soil water content effects on N_2_O emissions. All statistical analyses were performed using Genstat (version 17.0, VSN International Ltd., Hemel Hempstead, UK).

## 3. Results

### 3.1. Soil Physical and Chemical Properties

**Figure 1:**
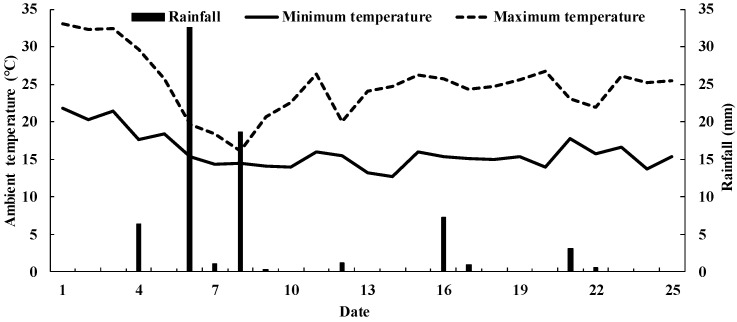
Daily ambient temperature and rainfall during the study.

**Figure 2:**
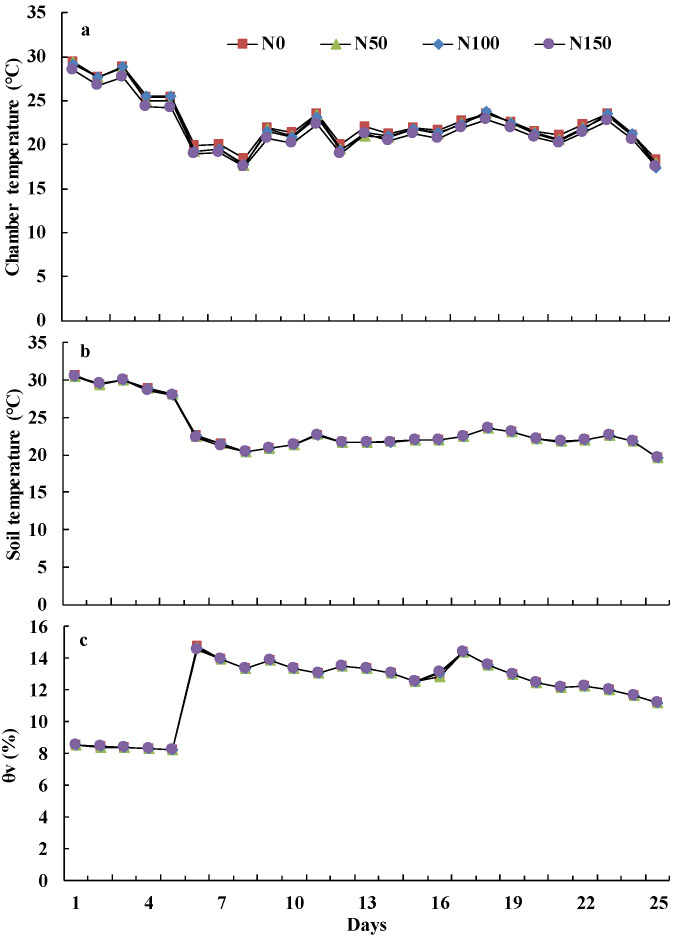
Average chamber temperature (**a**), 0–100 mm soil temperatures (**b**) and soil volumetric water content θ_v_ (**c**) from four nitrogen addition treatments.

Figure 1 shows daily ambient temperature including minimum and maximum temperatures and rainfall during the study. Figure 2 displays the chamber temperature (a), soil temperatures (b) and θ_v_ (c) of four treatments at a depth of 0–100 mm during the 25 days. There were no significant differences in mean chamber temperature among the treatments. Mean chamber temperature under N0, N50, N100 and N150 decreased significantly, by about 38%, 40%, 39% and 39% (*p* < 0.05), respectively, from Day 1 to 8 after three rainfall events, all exceeding 6.0 mm, respectively. The biggest decrease in chamber temperature occurred when the precipitation reached 32.6 mm and the temperature declined about 23% from Day 5 to 6 (*p* < 0.05).

Differences among mean soil temperatures of the four treatments at a depth of 0–100 mm were lower than those among chamber temperatures, and the trends in soil temperatures were similar to those of chamber temperatures (Figure 2a,b). The range of soil temperatures of each treatment was from 19.65 °C to 30.59 °C during the 25 days. Soil temperature declined about 20% from Day 5 to 6 when the precipitation accumulation was 32.6 mm (*p* < 0.05).

The range of θ_v_ of the four treatments at a depth of 0–100 mm was from 8.22% to 8.54% between Day 1 and 5. The largest θ_v_ value occurred after a 32.6 mm rainfall event, and the content increased by about 78% from Day 5 to 6 (*p* < 0.05). The range of mean θ_v_ values for each treatment was from 11.22% to 14.77% between Day 6 and 25.

**Figure 3:**
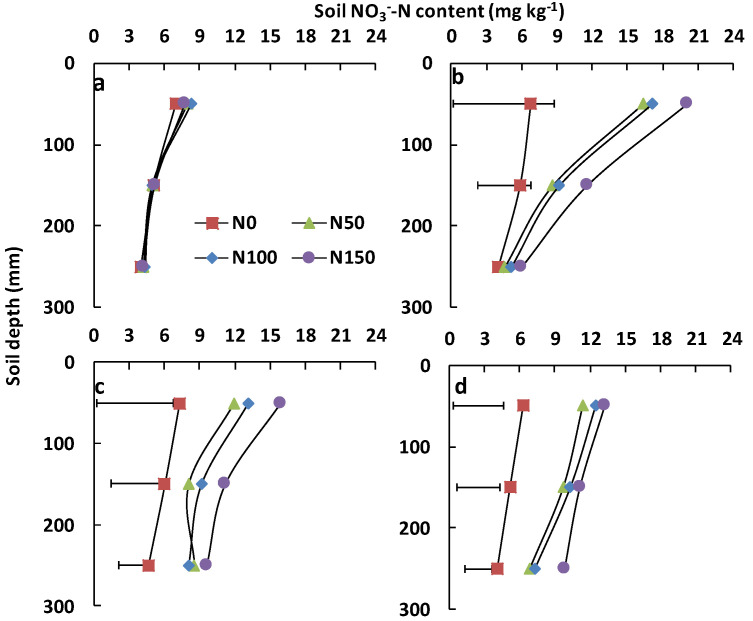
0–300 mm soil NO_3_^−^-N content from four nitrogen addition treatments before first (**a**), second fertilize (**b**), beginning (**c**) and end of experiment (**d**). The error bar indicated the least significant difference (LSD) *(p = 0.05)* of soil NO_3_^−^-N content between four nitrogen levels at each layer, *n* = 3.

Soil NO_3_^−^-N contents were lower than 8.34 mg kg^−1^ before the first fertiliser application (Figure 3a); fertiliser application increased the mean concentrations of NO_3_^−^-N contents in the 0–300 mm soil layer, especially after the first fertiliser application when 80% of the fertiliser was applied (Figure 3b). NO_3_^−^-N contents decreased with soil depth, and the values of NO_3_^−^-N contents increased with the fertiliser application amount. Compared to the N0 treatment, fertiliser application significantly increased the 0–100 mm soil NO_3_^−^-N content before the second fertiliser application (Figure 3b, *p* < 0.05); The 0–100 mm soil NO_3_^−^-N content under the N50, N100 and N150 treatments was 141%, 151% and 194% higher, respectively than that under N0 (Figure 3c, *p* < 0.05). The 200–300 mm soil NO_3_^−^-N content under the N50, N100 and N150 treatments were significantly higher than that under N0 (Figure 3c, *p* < 0.05). Soil NO_3_^−^-N contents in the 0–100 mm soil layer for the N0, N50, N100 and N150 treatments were 7.26, 11.91, 13.18 and 15.93 mg kg^−1^, respectively, at the end of the experiment (Figure 3d).

### 3.2. Grassland Yield, Height and Leaf Area Index

Fertiliser application did not significantly promote DM yield, height or light use efficiency of lucerne, although positive trends were present (Table 2). Before the experiment was conducted, the DM yields of the N50, N100 and N150 treatments were 5%, 5% and 8% higher than that of N0, respectively. The DM yields of the N50, N100 and N150 treatments were 6%, 10% and 10% greater than that of N0, respectively, at the end of the experiment. The maximum fertiliser application increased lucerne height by 16% before the experiment, from 504.5 mm to 584.4 mm, and by 6% at the end of the experiment, from 284.7 mm to 302.3 mm. Before experimenting, the range of LAI of each treatment was from 2.73 to 3.11. The range of LAI of each treatment was from 1.88 to 2.09 at the end of the experiment.

### 3.3. Soil N_2_O Emissions and Diurnal Variability

**Figure 4:**
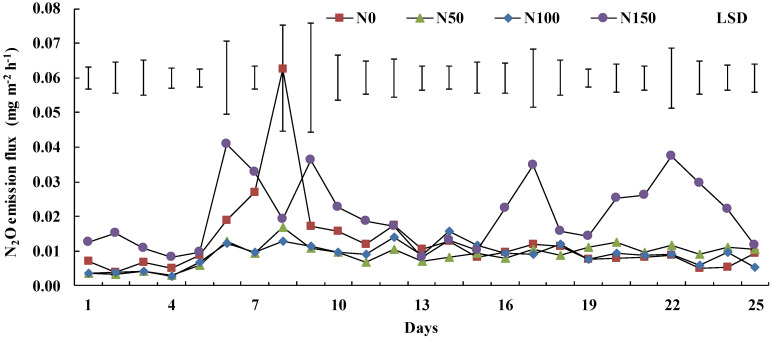
Mean N_2_O emission from four nitrogen addition treatments during the experiment period. Bars represent the LSD *(p = 0.05)* values between four nitrogen levels on each day, *n* = 3.

Mean N_2_O emission of the N0, N50, N100 and N150 treatments during the 25 days was 0.0127 ± 0.0014 (mean ± standard deviation), 0.0089 ± 0.0032, 0.0088 ± 0.0033 and 0.0206 ± 0.0089 mg m^−2^ h^−1^, respectively (Table S1, Figure 4). Nitrogen addition enhanced soil N_2_O emissions, and overall mean N_2_O emissions under the N150 treatment were 61% higher than those under the N0 treatment (*p* < 0.05). Daily N_2_O emissions were low under the N0, N50 and N100 treatments, but emissions under each treatment increased sharply on Day 6 following a 32.6 mm rainfall event, with emissions of 0.0187, 0.0127, 0.0123 and 0.0409 mg m^−2^ h^−1^, respectively. Emissions under the N0 treatment increased continually from Day 3 to 8, which included four periods of precipitation, ranging from 0.0066 to 0.0626 mg m^−2^ h^−1^. Emissions increased sharply from 0.0102 to 0.034 mg m^−2^ h^−1^ between Day 15 and 17 following a 32.6 mm rainfall event under the N150 treatment and increased again on Day 21 and 22, ranging from 0.0261 to 0.0374 mg m^−2^ h^−1^. While emissions increased slightly from 0.0051 to 0.0087, 0.0030 to 0.0058, 0.0028 to 0.0067 and 0.0081 to 0.0097 mg m^−2^ h^−1^ between Day 4 and Day 5 following a 6.3 mm rainfall event under N0, N50, N100 and N150, respectively. Emissions decreased under the N50, N100 and N150 treatments on Day 7, following a 32.6 mm rainfall event.

N_2_O emissions were significantly affected by the nitrogen addition amount (*p* < 0.001) and rainfall (*p* < 0.001, Table 3). Furthermore, there was an interaction effect between nitrogen addition and rainfall on the N_2_O emissions (*p* < 0.001).

**Figure 5:**
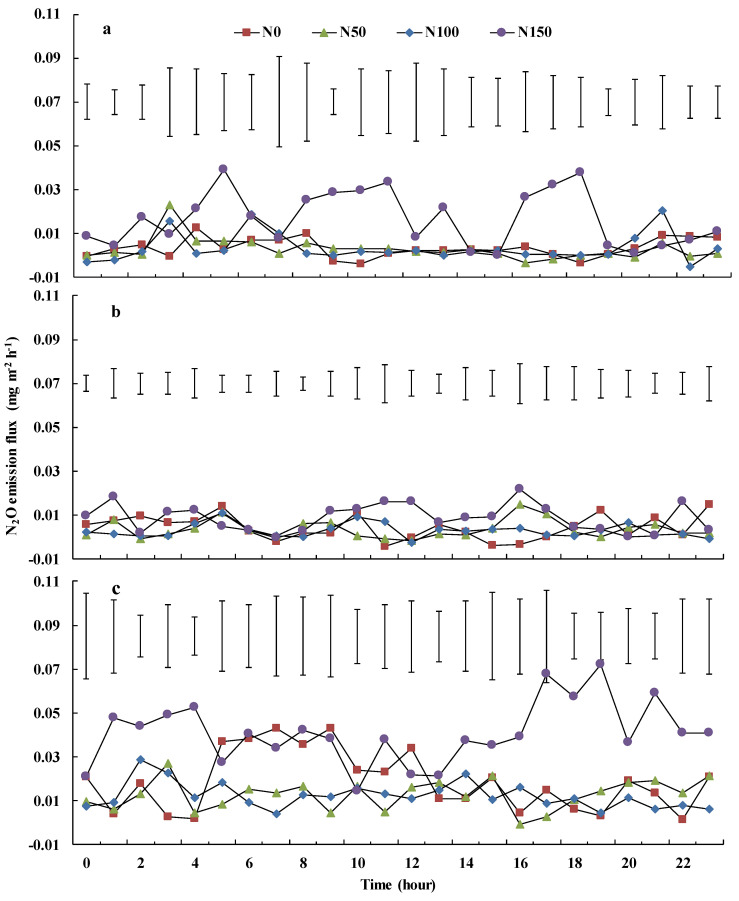
N_2_O emission daily dynamics of four nitrogen addition treatments on Day 2 (**a**), Day 4 (**b**) and Day 6 (**c**). Bars represent the LSD (*p* = 0.05) values between four nitrogen levels on each hour, *n* = 3.

The variability of N_2_O emissions after precipitation was different under each treatment, especially on Day 4 when the first rain occurred, after a more than a 30-day dry period, and on Day 6, when the highest rainfall occurred. We chose Day 2 as a representative dry period. The daily variability of N_2_O emissions was highest for N150 on Day 2, with a minimum of 0.0001 mg m^−2^ h^−1^ and a maximum of 0.0394 mg m^−2^ h^−1^ (Figure 5a). The mean N_2_O emission under N150, N0, N100 and N50 treatments, in descending order, were 0.0167, 0.0035, 0.0034 and 0.0029 mg m^−2^ h^−1^, respectively. The daily dynamic trend in N_2_O emissions declined first and then increased under N0, N50 and N100 treatments.

Except from 0900 to 1200 and 1500 to 1700, the diurnal variability of N_2_O emissions was stable in all four treatments on Day 4, when a 6.3 mm rainfall event occurred (Figure 5b). Flux was 0.0044 ± 0.0056 (mean ± standard deviation), 0.0036 ± 0.0042, 0.0029 ± 0.0032 and 0.0086 ± 0.0063 mg m^−2^ h^−1^ under N0, N50, N100 and N150, respectively.

Diurnal variability of N_2_O emissions was stable under the N50 and N100 treatments on Day 6 when a 32.6 mm rainfall event occurred (Figure 5c). Daily variation in N_2_O emission was highest under N150, with a minimum of 0.0142 mg m^−2^ h^−1^ and a maximum of 0.0720 mg m^−2^ h^−1^. The daily dynamic of fluxes increased first and then declined under the N0 treatment on Day 6 and under the N100 treatment decreased at first and then increased.

### 3.4. Relationships between Soil N_2_O Emissions and Soil Water Content

**Figure 6:**
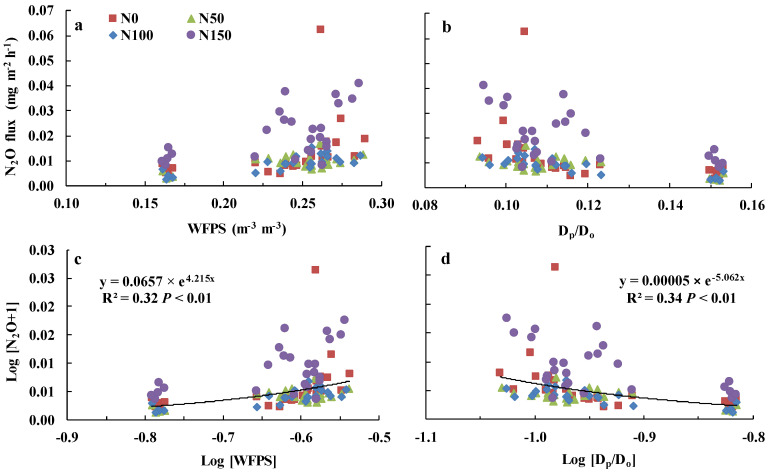
Mean N_2_O emission and (**a**) water-filled pore space (WFPS) and (**b**) relative soil diffusivity (D*_p_*/D*_o_*); Exponential regression of mean Log [N_2_O + 1] and (**c**) Log [WFPS] or (**d**) Log [D*_p_*/D*_o_*] from four nitrogen addition treatments, data points are individual replicates (*n* = 3).

N_2_O emissions were highest when WFPS values were between 0.23 and 0.29 m^−3^ (Figure 6a) and when D*_p_*/D*_o_* values were between 0.09 and 0.12 (Figure 6b). Pooling all N_2_O flux data from the four treatments and performing exponential regression analysis between log-transformed WFPS or D*_p_*/D*_o_* and log-transformed daily N_2_O emissions showed that D*_p_*/D*_o_* better explained the variation in daily N_2_O emissions (Figure 6c,d).

## 4. Discussion

### 4.1. Soil Nitrogen Dynamics

Soil NO_3_^−^-N content increased after nitrogen fertiliser addition, particularly in the 0–100 mm soil layer (Figure 3b). While the 100–300 mm soil NO_3_^−^-N content was not significantly different between the N0 and N50 or N100 treatments, this could be explained by lucerne accelerating uptake of mineralized nitrogen after the first harvest removed a substantial amount of aboveground biomass [32]. Soil NO_3_^−^-N content in the 200–300 mm soil layer under the N50, N100, and N100 treatments increased at the beginning of this study (Figure 3c), which might have been caused by precipitation and subsequent leaching [8,14]. With further rainfall events during the experiment, soil NO_3_^−^-N content in the 0–100 mm soil layer under the N50, N100, and N100 treatments declined at the end of the experiment (Figure 3d). Rainfall disturbs topsoil structure and accelerates soil mineralization by influencing soil water content and temperature; these factors will enhance nitrogen loss by gas emissions [33].

### 4.2. Soil N_2_O Emissions and Diurnal Variability

Although N_2_O emissions were significantly affected by rainfall and nitrogen addition (Table 3), the relationship between soil NO_3_^−^-N content and N_2_O emissions was not obvious (Figure S1). Topsoil NO_3_^−^-N content, as the direct substrate of N_2_O [34], did not significantly differ between the N50 and N100 treatments (Figure 2b), which is a potential explanation. Especially after Day 16, N_2_O emissions under N50 and N100 treatments were identical (Figure 1 and Figure 4), and at the same time, the difference in the 0–100 mm soil NO_3_^−^-N content between treatments declined too (Figure 3c). More N_2_O was released under N150 because that high-concentration fertiliser application increased soil nitrogen concentration (Figure 2b,c). Normally, enhanced N_2_O emissions from agricultural ecosystems are believed to be driven by increased fertiliser application [1]. The observation that nitrogen addition did not enhance N_2_O emissions significantly was in agreement with the findings of Shcherbak et al. (6), who suggested that modest nitrogen addition would have little impact on N_2_O emissions. Additionally, legume–*Rhizobium* symbioses could produce N_2_O as a result of rhizobia denitrification [35], but rhizobia are acutely sensitive to nitrogen addition. Nitrogen fertiliser can restrict rhizobia activity or nodulation [36] and, thus, restrict N_2_O emission via denitrification.

Soil temperature affects N_2_O emissions by influencing metabolic activities of nitrifiers or denitrifiers and substrate availability [37]. Differences in soil temperature between each treatment could also explain N_2_O dynamics, such as those observed on Day 6 (Figure 2b and Figure 5c). However, this is not a strong explanation for the daily dynamics of N_2_O emissions, especially on Day 4, when the daily dynamics of N_2_O were relatively stable (Figure 5b). Soil water content alters N_2_O emissions through soil aeration [37]. Change in soil water content between each treatment could well explain the N_2_O dynamics of Day 6, Day 8 (Figure 2b and Figure 5) and Day 17. Additionally, increased soil water content hampered gas diffusion and reduced N_2_O emissions [38]. Thus, low variability in N_2_O emissions on Day 4 was likely caused by a 6.3 mm rainfall event, which did not change water content significantly, but might alter the aeration of topsoil, or may even have restricted N_2_O emission after a 32.6 mm rainfall event (Figure 2, Figure 5 and Figure 6b). N_2_O emissions also largely varied under N150 treatment over 16–25 days, which indicates that apart from soil water and NO_3_^−^-N content, and soil temperature, there might be other variables (e.g., activities of enzymes and microbial) that affect N_2_O emission, and further study is warranted.

### 4.3. Relationship of N_2_O with WFPS and D_p_/D_o_

With the increase of WFPS and decline of D*_p_*/D*_o_*, mean N_2_O flux increased significantly (Figure 6a,b). Although log-transformed D*_p_*/D*_o_* was able to explain slightly more of the variation in log-transformed N_2_O emissions than log-transformed WFPS (Figure 6c,d), D*_p_*/D*_o_* suggests a critical value, 0.006, as a threshold for N_2_O production and consumption [23,25,27]. Soil D*_p_*/D*_o_* can relate N_2_O emissions to changes in soil water content even under different soil matrix potential and bulk density values [23,24,25,26,27]. Additionally, D*_p_*/D*_o_*, which integrates soil porosity and pore size distribution, is a good predictor of soil O_2_ supply [39]. WFPS could explain variation in N_2_O flux magnitude with changes in soil water content [13,16,17], but despite this, WFPS cannot represent the fraction of the entire soil volume and soil pore connectivity or tortuosity [16,19], Farquharson and Baldock (19) even suggested that WFPS could be replaced with θ_v_ when estimating N_2_O emissions across soils that vary in bulk density.

Harrison-Kirk et al. (27) observed that the relationship between total nitrogen (N_2_O + N_2_) flux and D*_p_*/D*_o_* was stronger than that between D*_p_*/D*_o_* and N_2_O flux. Balaine (25) found that D*_p_*/D*_o_* reflects both N_2_O and N_2_ emissions as well. Because our study did not measure N_2_ flux, D*_p_*/D*_o_* may have appeared superior to WFPS in explaining N_2_O emissions of lucerne grassland, which also demonstrates the importance of measuring total nitrogen (N_2_O + N_2_) flux. Nitrification might be the largest source of N_2_O emissions in semi-arid regions, where soils are rarely under anaerobic conditions that promote denitrification [13,40,41]. The range of 0–100 mm soil θ_v_ values in the current study was 8.22–14.77% (Figure 2c); thus, nitrification and not denitrification was likely the main source of N_2_O emissions, and this also was observed in our study as high D*_p_*/D*_o_* values (Figure 6b). These findings indicate that how D*_p_*/D*_o_* relates N_2_O emissions to changes in soil water content when nitrification dominates the production of N_2_O needs to be confirmed in future research.

Owens (39) found that values of D*_p_*/D*_o_* smaller than 0.10 are exponentially, rather than linearly, related to WFPS, and D*_p_*/D*_o_* declines with increases in WFPS. Low θ_v_ at a depth of 0–100 mm led to higher D*_p_*/D*_o_* values, ranging from 0.09 to 0.15 (Figure 2b), and log-transformed D*_p_*/D*_o_* was linearly negative correlated with log-transformed WFPS (*Y* = −0.82*x* − 1.45, *R*^2^ = 0.99), which was also consistent with D*_p_*/D*_o_* being just slightly better than WFPS in explaining the variation in N_2_O emissions (Figure 6c,d).

Negative N_2_O emissions occurred unexpectedly in the study (Figure 4 and Figure 5). Negative N_2_O emissions usually indicate that soil N_2_O was reduced and N_2_ was emitted, as could occur under anaerobic conditions, low mineral N and readily available organic matter as energy sources for denitrifying microbial activity [9,34]. Barton et al. (13) hypothesized that soils with low water content were rarely sufficiently anaerobic to trigger denitrification. Furthermore, the emergence of negative N_2_O emissions also partly influenced the relationship between D*_p_*/D*_o_* and N_2_O emissions in the lucerne grassland plots. However, Chapuis-lardy et al. (2007) suggested that negative N_2_O fluxes would not be a common phenomenon. Particularly, the study was performed over a time period when N_2_O uptake occurred unexpectedly.

## 5. Conclusions

Nitrogen addition and rainfall significantly affected N_2_O emissions, while the N_2_O emission response to increasing nitrogen input was not linear. Soil D*_p_*/D*_o_* and WFPS were good indicators of N_2_O diurnal dynamics, and D*_p_*/D*_o_* was able to explain slightly more of the variation in N_2_O emissions than WFPS, even when θ_v_ was less than 20 m^−3^. Thus, more research should be focused on D*_p_*/D*_o_*, which could be a better proxy for N_2_O emissions, even when both rainfall and the addition of nitrogen strongly shape variability in N_2_O emissions.

## Figures and Tables

**Table 1:** Soil carbon and nitrogen contents of the lucerne grasslands in different depths. (Mean ± SD, *n* = 4).

Layer (mm)	SOC (mg g^−1^)	TN (mg g^−1^)	C/N
0–100	10.23 ± 1.61	1.12 ± 0.35	9.13 ± 1.70
100–200	8.53 ± 0.87	0.88 ± 0.21	9.74 ± 2.19
200–300	7.54 ± 0.63	0.88 ± 0.14	8.61 ± 1.14

SOC represents soil organic carbon; TN represents total N.

**Table 2:** Dry matter (DM) yield, height and leaf area of each treatment before (**A**) and at the end of experiment (**B**). (Mean ± SD, *n* = 3).

	Treatment	DM Yield (kg ha^−1^)	Height (mm)	LAI
**A**	N0	4635.93 ± 221.76	504.4 ± 26.5	2.73 ± 0.20
	N50	4855.19 ± 537.69	551.1 ± 42.3	2.95 ± 0.32
	N100	4848.89 ± 401.11	581.1 ± 36.9	3.06 ± 0.27
	N150	4985.93 ± 396.86	584.4 ± 50.8	3.11 ± 0.26
**B**	N0	3281.56 ± 270.76	284.7 ± 20.5	1.99 ± 0.53
	N50	3490.23 ± 467.65	318.4 ± 35.5	1.88 ± 0.42
	N100	3622.83 ± 578.46	299.7 ± 25.9	2.09 ± 0.37
	N150	3594.97 ± 933.39	302.3 ± 33.7	1.88 ± 0.39

**Table 3:** Two-way ANOVA for the effects of nitrogen addition amount (N) and rainfall (R) on N_2_O emission, *n* = 30.

Factor	Sum of Squares	df	Sum of Squares	*F*	*p*
N	0.008	3	0.003	112.68	<0.001
R	0.005	9	0.001	24.41	<0.001
N × R	0.010	27	<0.001	14.38	<0.001

## Data Availability

Data are available from the author on request.

## Supplementary Materials

The following supporting information can be downloaded at: https://www.mdpi.com/article/10.3390/ijerph19137789/s1. Table S1. Average and cumulative N_2_O emissions from four nitrogen addition treatments during the experiment period.
