# Supplementary material for: Nitrogen Addition Affects Nitrous Oxide Emissions of Rainfed Lucerne Grassland
Source: Int J Environ Res Public Health. 2022 Jun 24;19(13):7789. doi: 10.3390/ijerph19137789 (PMC9265669; doi:10.3390/ijerph19137789)
Supplement: Supplementary file 1 [file ijerph-19-07789-s001.zip › ijerph-1736790-supplementary.pdf]

Table S1. Average and cumulative N_2O emissions from four nitrogen addition treatments during the experiment period.

	**Mean N_2O Emissions** **mg m^{-2} h^{-1}**	**Cumulative N_2O Emissions** **g N m^{-2}**
N0	0.0127±0.0014 b	0.0049±0.0002 b
N50	0.0089±0.0032 c	0.0034±0.0007 c
N100	0.0088±0.0033 c	0.0034±0.0008 c
N150	0.0206±0.0098 a	0.0079±0.0011 a

Note: Data shown are mean ± standard deviation, n = 3. Values were compared using ANOVA and letters represent homogeneous groups obtained from post-hoc analysis (Tukey honest significant difference [HSD] test). Significance levels are given for differences between sites ($p < 0.05$) and means denoted by different letters.
